# Experimental Investigation of Current Intensity and Feed Speed in Electrically Assisted Necking and Thickening of 5A02 Aluminum Alloy Tubes

**DOI:** 10.3390/ma17040771

**Published:** 2024-02-06

**Authors:** Yubin Fan, Xuefeng Xu, Ruichen Tao, Ming Luo, Xiaodong Li, Liming Wei, Shitian Wu, Jie Xiao, Xiang Zeng

**Affiliations:** 1 School of Aviation Manufacturing Engineering, Nanchang HangKong University, Nanchang 330063, China; yubin_fan@hotmail.com (Y.F.); taoruichen98@163.com (R.T.); xiaodong.li@mail.hfut.edu.cn (X.L.); 71153@nchu.edu.cn (L.W.); 33025@nchu.edu.cn (J.X.); zengxiang@nchu.edu.cn (X.Z.); 2 Key Laboratory of High Performance Manufacturing for Aero Engine, Ministry of Industry and Information Technology, Northwestern Polytechnical University, Xi’an 710072, China

**Keywords:** necking and thickening, electrically assisted forming, current intensity, thin-wall tube, process window

## Abstract

In order to further explore the forming limits of thin-wall tube necking and thickening, and obtain sufficient thickness of the tube in the thickening area, local electric pulse-assisted forming experiments were carried out to study the effects of current intensity and feed speed on the necking and thickening forming of thin-wall tube. The experimental results show that with the increase in current intensity, the temperature in the forming area of the tube increases, and the forming load for necking and thickening decreases. However, with the increase in feed speed, the overall forming load for necking and thickening increases in general, and the smaller feed speed is more conducive to forming. Taking into account the forming efficiency and electrode loss, the corresponding forming process window is obtained for the manufacturing of good parts. That is, during the necking stage, the current intensity shall not be less than 300 A, and the feed speed shall not exceed 10 mm/min. During the thickening stage, the current intensity should not be less than 1400 A, and the feed speed should not exceed 1 mm/min. The target part is finally formed, with an average wall thickness of 5.984 mm in the thickening zone and a thickening rate of 303.2%.

## 1. Introduction

Government regulations and environmental standards have pushed design and manufacture toward high energy efficiency, high stability, and high life of equipment, particularly in the aviation and aerospace industries. Therefore, the requirements of lightweight and high performance are also put forward for the parts and components involved. The aviation pull rod is one of the most important components of aircraft control systems. Traditional aviation pull rods need to be connected by welding or riveting a threaded sleeve at the straight end of the tube necking zone. If the straight end area of the tube necking zone has sufficient thickness to tap the thread, the sleeve can be omitted, which will help improve the connection strength and achieve the lightweight of the pull rod. Due to the poor formability of hard-to-form metals such as aluminum alloy at room temperature, it is difficult to achieve thickening for the straight end of the tube necking zone. In the current research, thermally assisted forming [1,2,3] and electrically assisted forming [4,5,6] have been proven to be effective means in terms of reducing forming flow stress and improving formability for different hard-to-deform alloys.

Through the introduction of hot forming into the corresponding tube forming, such as flaring, spinning, extrusion, etc., the existing studies have shown that the formability can be effectively improved. The end flaring of the commercially pure grade 2 titanium tube is experimentally and numerically investigated at room and high temperatures [7]. It is shown that cylindrical, elliptical, and square flaring with specified dimensions, which are not possible at room temperature, can be successfully carried out at a temperature of 400 °C. For the hot spinning of titanium alloy, Gao et al. [8] revealed the effects of process parameters and microstructure on damage evolution. Among them, with the increase in initial spinning temperature and roll fillet radius, the degree of damage gradually decreases, which is beneficial for forming. To overcome the limits of traditional room temperature processes, an innovative rotary draw bending process carried out at high temperatures is proposed to increase the material’s formability and obtain the target microstructure in the final part [9]. On this basis, Chen et al. [10] further explored the effects of the laves phase on the burst behavior of GH3625 nickel-base superalloy pipe during hot extrusion. In order to realize the thickening for the straight end of the pull rod necking zone, we proposed a new method, which is to achieve necking and thickening of the aluminum alloy thin-walled tube through differential temperature extrusion [11]. The effects of temperature and friction on forming were conducted through finite element simulation and experiments. Although the straight end of the tube necking zone has been thickened, its thickness is still relatively thin.

Compared to thermal-assisted forming, electric-assisted forming exhibits greater advantages in reducing the required deformation energy and increasing the metal’s formability through the use of electrical current [12,13,14]. In many cases, previous work has shown that electrically assisted forming has resulted in metals being formed further than conventional forming methods alone without sacrificing strength or ductility. The contribution in the flow stress reduction from heating by an external source was less than that from tension with pulse current at the same temperatures [15,16]. Mohammadtabar et al. [17] focused on the effect of the electric current pulse type on the springback, microstructure, texture, and mechanical properties during the V-bending process of the AA2024 aluminum alloy. The electric pulses improved the formability of the AA2024 alloy owing to the activation of more slip systems, the inhibition of dislocation pinning, the promotion of dislocation movement, and the acceleration of restoration mechanisms. Further, the effect of the electric current path on force drop and springback was studied by carrying out experiments and fully coupled electro-thermo-mechanical finite element analysis [18]. Results indicated that significantly higher force drop and springback reduction were observed when current was passed through the deformation zone compared to throughout the specimen. In addition, in terms of tube forming, Wagner et al. [19] incorporated electrically assisted forming into the microtube hydroforming to reduce the forces and pressures. Tests of annealed stainless steel 304 tubing have shown that the ultimate tensile strength and bust pressure decrease with increased current while using electrically assisted forming during microtube hydroforming. Wang et al. [20] developed three-dimensional finite-element modeling of electrically-assisted rotary-draw bending of 6063 aluminum alloy micro-tubes and studied the effects of diameter, bending radius, current density, and electrical load path on the bending defects of the Al6063 micro-tubes. Jiang et al. [21] present a high-current power supply for an electrically assisted three-roll incremental rolling system capable of producing tubes of various diameters without the need for die/tool replacement. Liu et al. [22] proposed an electrically assisted (EA) micro-forming process and investigated the deformation characteristics and performance evolution of capillaries under electric current to achieve the precise and efficient fabrication of capillaries. The above various electric-assisted forming processes indicate that introducing pulsed current can effectively improve the part quality. However, there are few reports on the introduction of electrical assistance in the forming process of aluminum alloy tube necking and thickening.

Our team has conducted preliminary research on electric-assisted tube necking and thickening in the early stage. Based on the design of the electric-assisted tube necking and thickening device, the device composition has been optimized in aspects such as the thickness of the guide block, the contact mode of the upper electrode, the cooling method, etc. [23]. However, the influence laws of key loading parameters still need to be further studied. Through preliminary experiments in the early stage, we found that current density and feed speed are the most important parameters affecting necking and thickening formation. In this article, further research is conducted on the influence laws of the two key parameters, current density, and feed speed. At the same time, we would establish a segmented optimization process for necking and thickening forming. The forming process is subdivided into the necking stage and the thickening stage, and the effects of current density and feed speed in these two stages will be investigated, respectively. Through experimental research, the forming process window of two stages is explored, respectively. This will provide a basis for manufacturing better necking and thickening pull rod parts, help achieve precise control in stages, reduce energy loss, and improve forming efficiency.

## 2. Experiment of Electrically Assisted Necking and Thickening

### 2.1. Electrically Assisted Necking and Thickening Principle

This experiment aimed to fabricate an aluminum alloy thin-walled tube into a necking and thickening tube. The initial 5A02 aluminum alloy tube was 2 mm in wall thickness and 22 mm in outer diameter. 5A02 aluminum alloy belongs to the Al-Mg series of high plasticity and high fatigue strength aluminum alloys, which have high corrosion resistance, good weldability, good cold workability, and forming workability. The relevant physical parameters of the 5A02 aluminum alloy are as follows: thermal conductivity is 156 W/(m·K), specific heat capacity is 947 J/(kg·K) and density is 2680 kg/m^3^.

**Figure 1:**
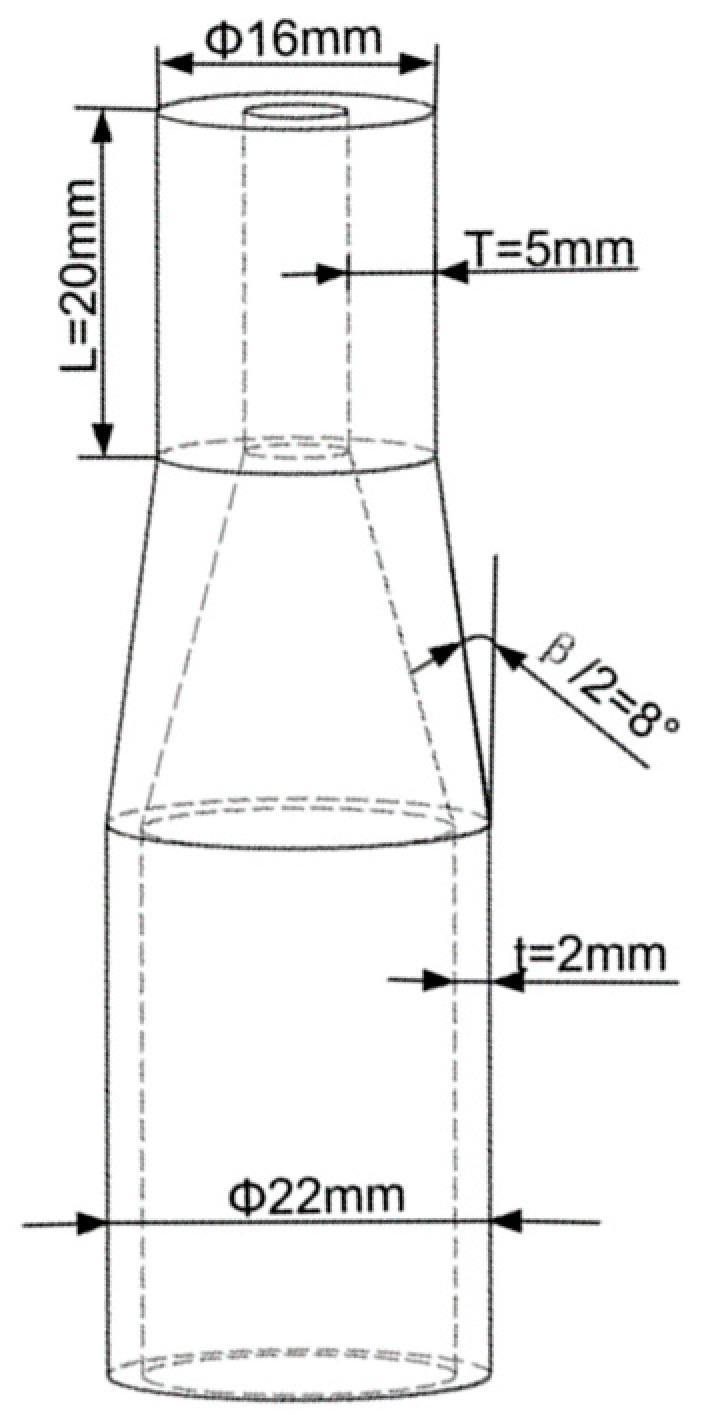
Target necking and thickening tube.

As shown in Figure 1, the dimensions of the acquired necking thickened tube were as follows: The initial wall thickness t of the straight end was 2 mm; the taper angle of the necking zone was 16°; while the thickening zone was 20 mm in length, Φ16 mm in outer diameter, and 5 mm in wall thickness T.

**Figure 2:**
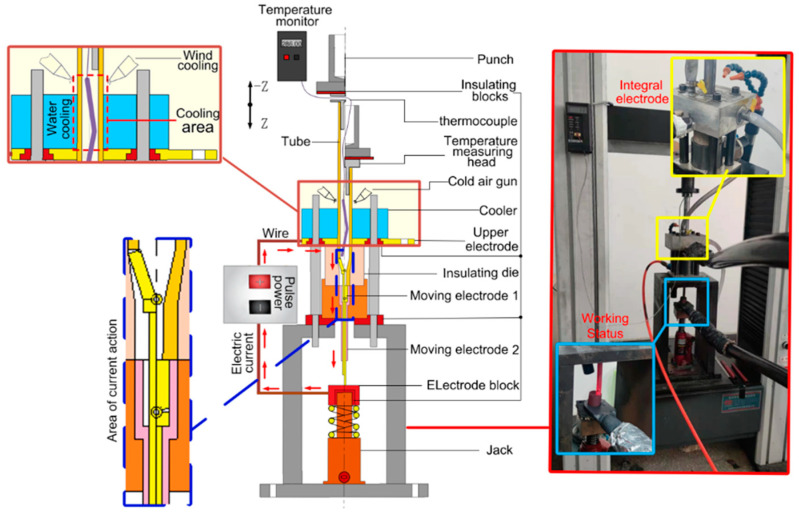
The local electric pulse-assisted necking and thickening device [20].

For this reason, our team independently developed a set of local electric pulse-assisted necking and thickening devices for the tube, as shown in Figure 2. The experimental device is mainly composed of a pusher, tube blank, cooler, electrode block, die, temperature measurement device, insulating block, and so on. The pusher is at the top of the device, connected to the universal testing machine, and transfers the load to the tube blank. The cooler (blue part in the figure) includes a cold air gun and a water cooling block to cool the force transfer area of the tube blank. The electrode block (yellow part in the figure) comprises an upper electrode block and a moving electrode, which are connected to the pulse power supply to ensure the current loading of the forming part of the tube blank during the forming process. Therein, the output voltage of the power supply is 8 V, the output current is up to 1500 A, and the current accuracy is accurate to 0.1 A. The loaded current is based on the output current set by the pulse power supply; the die (peach-colored part in the figure) is made of insulated alumina ceramic material to avoid excessive energy loss and ensure that the current flows through the tube blank during forming, and the temperature measurement device consists of a thermometer and a thermocouple. The thermocouple enters the interior of the tube blank through the hole on the side of the pusher to measure the temperature of the target area (in this experiment, a K-type armored thermocouple was selected, and the armored part was sealed with high-temperature epoxy resin adhesive. The probe diameter was 2 mm, which can be freely bent. The temperature measurement range was 0–1100 °C, with fast response speed and stable performance.); the insulating block (dark red part in the figure) prevents current from flowing into the electronic device and causing damage to the device.

The forming principle is described as follows: An electrifying circuit is formed through the connection of a pulsed power supply-upper electrode block-tube blank-moving electrode-conductive block. Therein, the tube blank’s part in the cavity of the punch die is electrified, and the tube temperature in the zone formed by this part rises under the action of the Joule thermal effect and electro-plastic effect, thus achieving better plasticity. Meanwhile, the local tube blank above the upper electrode block is cooled using a cooler to ensure the strength of the force transfer zone of the tube blank and avoid buckling of the tube. When the temperature reaches the specified value, the load is applied to the pusher through the universal testing machine, and the pusher feeds downward to finish the necking and thickening of the tube in turn with the control of pushing displacement. In the forming process, spring support is sleeved between the insulating block and the jack to ensure that the tube is continuously electrified by the power supply and the conductive block keeps contact with the moving electrode when descending.

### 2.2. Electrically Assisted Necking and Thickening Principle

The local electric pulse-assisted necking and thickening experiment of the 5A02 aluminum alloy tube was carried out using the developed device. To obtain a necking and thickening tube of good quality, the current intensity and feed speed in the necking and thickening stages were studied. The specific experimental scheme is shown in Table 1:

## 3. Experimental Results and Analysis

### 3.1. Effect of Current Intensity

#### 3.1.1. Effect of Current Intensity on Necking Forming

**Figure 3:**
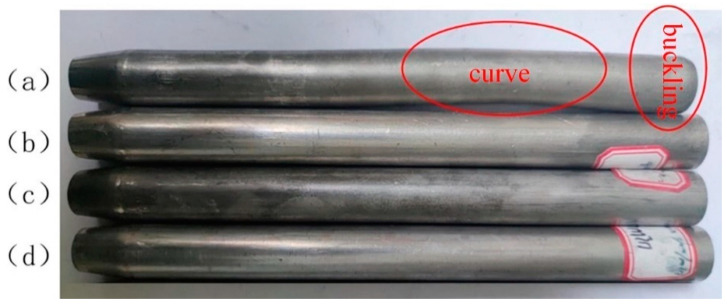
Necking parts of the 5A02 aluminum alloy tube under different current intensities: (**a**) I = 100 A; (**b**) I = 200 A; (**c**) I = 300 A; (**d**) I = 400 A.

Figure 3a shows the necking tube formed under the current intensity of 100 A. After the necking forming was completed, evident buckling occurred to the contact part between the tube and the pusher in the tube supporting zone during the forming process.

This is because the current intensity of 100 A produces less heat for the aluminum alloy tube with small resistance, and the deformed end of the tube is low, accompanied by insufficient plasticity. In the case of necking deformation, when the forming load exceeds the acceptable limit of the tube supporting zone, the contact part between the tube and the pusher will buckle first, and with the continuous increase of the load, the tube in the supporting zone will be subjected to instability phenomena like buckling. Figure 3b shows the necking tube formed under the current intensity of 200 A, which displayed good forming quality without buckling or wrinkling after necking forming. The conditions of the necking tube formed under 300 A and 400 A were basically identical to those in Figure 3b, namely, the forming quality was good after necking forming. Hence, the necking deformation of the tube could be well realized under the current intensity of 200–400 A.

**Figure 4:**
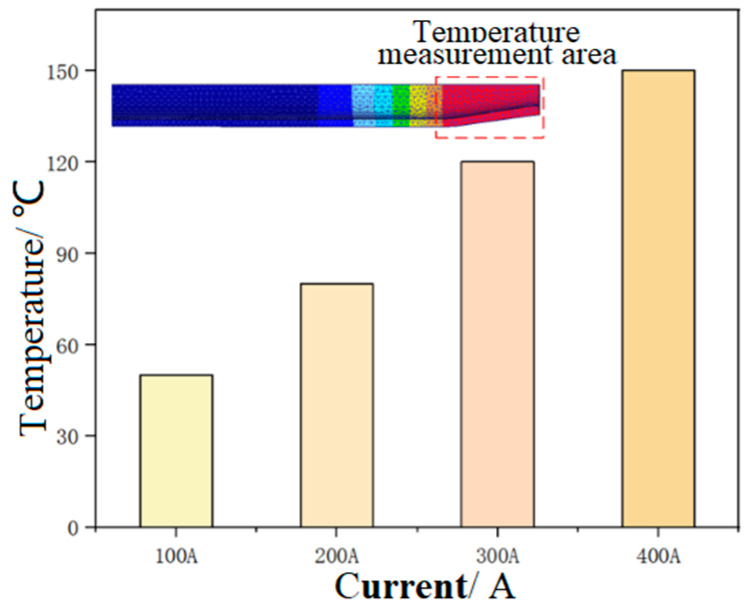
The necking temperature of the tube under different current intensities.

**Figure 5:**
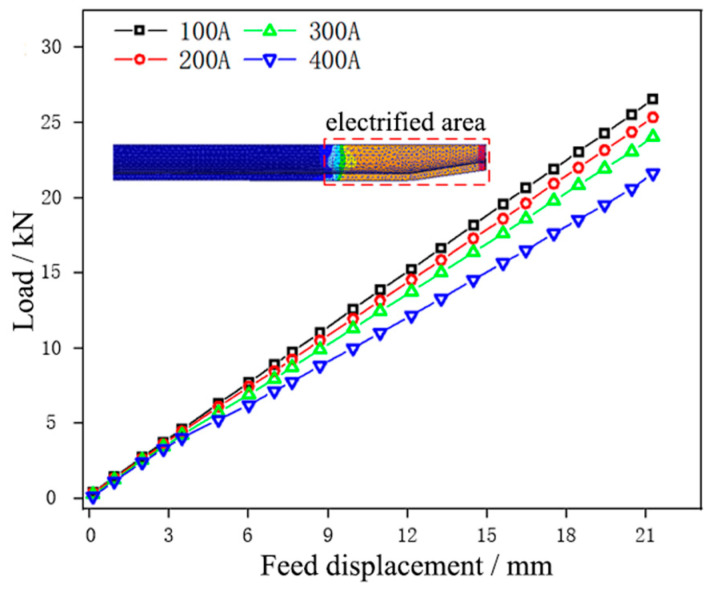
Displacement-load curves of the tube under different current intensities.

Figure 4 and Figure 5 show the forming temperature and load of the tube under different current intensities, respectively.

It could be seen that the temperature of the tube blank in the necking zone increased with the increase of current intensity, and the forming load showed an opposite trend. Under the current intensity of 100 A, the temperature in the necking zone of the tube blank was the lowest, being 53 °C. In this case, the tube blank displayed poor plasticity, and the load required for necking forming was the highest; under the current intensity of 400 A, the temperature in the necking zone of the tube blank was the highest at 147 °C. In this case, the plasticity of the tube blank was the highest, and the forming load required for necking was the lowest. The results reveal that a large current intensity can reduce the load and contribute more to forming during tube necking. To avoid damage to the electrode die, the current intensity in the necking stage was chosen as 300 A.

#### 3.1.2. Effect of Current Intensity on Thickening Forming

The effect of current intensity on the thickening forming of 5A02 aluminum alloy tube fittings was analyzed through the control variable method, as shown in Table 1, and the forming tests with current intensities of 800 A, 1000 A, 1200 A, and 1400 A were carried out, respectively.

**Figure 6:**
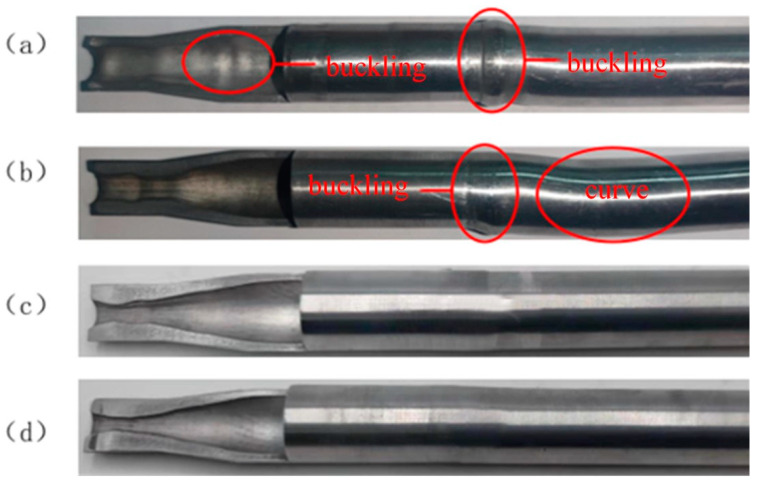
Thickening parts of the 5A02 aluminum alloy tube under different current intensities: (**a**) I = 800 A; (**b**) I = 1200 A; (**c**) I = 1200 A; (**d**) I = 1400 A.

Figure 6a shows the thickened tube fitting formed under the current intensity of 800 A.

The straight section of the tube blank was formed, the thickening forming was not obvious, and buckling occurred in both the necking zone and the supporting zone of the tube blank. This is because the heat generated by the current intensity of 800 A fails to meet the conditions of tube blank thickening, the temperature is relatively low, the temperature gradient difference between the tube blank thickening zone, necking zone, and supporting zone is not large enough, the tube blank in the thickening zone is not softened enough, the load required for thickening forming is large, the initial load is close to the bearing limit of the tube blank’s supporting zone, and buckling occurs easily. When the thickening load of the tube blank exceeds the bearing limit of the supporting zone, the tube blank will buckle, and the thickening process has just begun. Figure 6b shows the thickened tube formed under the current intensity of 1000 A. After the straight section forming was completed, the thickening forming achieved a certain effect. The wall thickness of the thickening zone was great, failing to meet the target requirements, and the tube blank’s supporting zone buckled and bent. Although the heat generated by the current intensity of 1000 A was higher than that of 800 A, the temperature gradient difference in the thickening zone, necking zone, and supporting zone of the tube blank increased, and the softening effect of the tube blank in the thickening zone was more evident. However, the temperature was still not high enough, the thickening forming load was still large, and the tube blank was prone to instability phenomena like buckling. Figure 6c shows the thickened tube fitting formed under the current intensity of 1200 A. It could be observed that the thickening forming effect was apparent after straight section forming. The wall thickness of the thickening zone met the target requirements, and the tube fitting was kept from buckling, bending, and other instability phenomena. This is because when the current intensity reaches 1200 A, the temperature of the tube blank reaches the required temperature for thickening, and the temperature gradient difference between the thickening zone, the necking zone, and the supporting zone of the tube blank is large enough, the softening effect of the tube blank in the thickening zone is obvious, and the initial forming load of thickening is far below the bearing limit of the supporting zone of the tube blank, which is enough for thickening and forming the tube blank to a certain extent. Figure 6d shows the thickened tube fitting formed under the current intensity of 1400 A. The best thickening forming effect was achieved after the straight section forming was completed, accompanied by the greatest wall thickness of the thickening zone, and the tube fitting was free from buckling, bending, and other instability phenomena. Therefore, the current intensity suitable for tube thickening should not be less than 1200 A.

**Figure 7:**
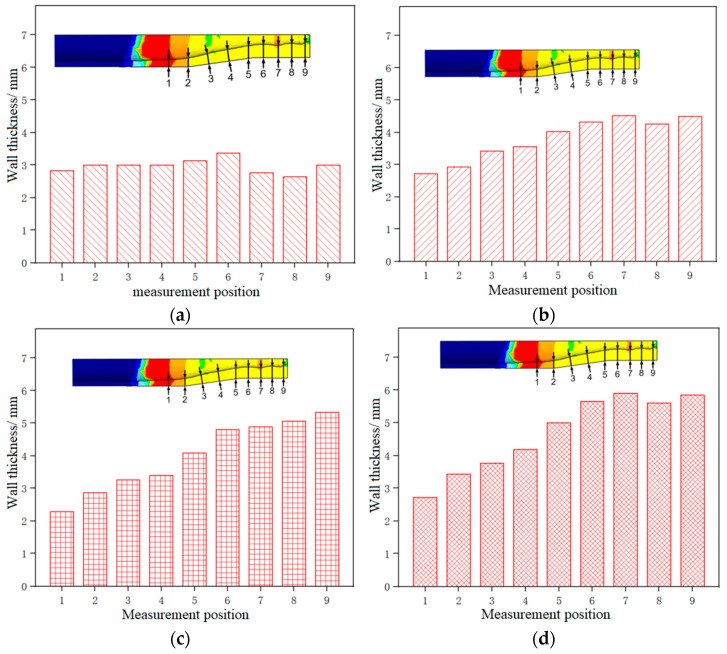
Wall thickness distribution of 5A02 aluminum alloy tube thickening parts under different current intensities: (**a**) I = 800 A; (**b**) I = 1000 A; (**c**) I = 1200 A; (**d**) I = 1400 A.

Figure 7 shows the wall thickness distribution of the thickened tube formed under different current intensities. For each parameter condition, three repeated experiments were conducted, and the results were analyzed based on the average wall thickness of the three experiments. Among them, the wall thickness at different positions is measured by cutting the specimen and using a vernier caliper.

It could be seen that the thickness of the thickening zone of the tube fitting was the largest, while the thickness of the supporting zone was the smallest, and the wall thickness increased from the supporting zone to the thickening zone. As the current intensity increased from 800 A to 1400 A, the thickness of the thickening zone gradually increased, and the thickness of the supporting zone generally showed a downward trend, indicating that the greater the current intensity, the higher the temperature of the tube blank, the better the material flow performance, the more obvious the electro-plastic effect, the more the materials flowing to the thickening zone, and the less the materials accumulated in the supporting zone. The thickness at the end of the supporting zone (Point 1) was the smallest under the current intensity of 1200 A, and the largest under 800 A, and the thickening rate was 114% and 142%, respectively. This is because when the current intensity is 1400 A, the plasticity of the tube blank is obviously higher than that of the tube blank under the current intensity of 1200 A, and it can bear a larger total amount of feed, and a small quantity of materials will accumulate at Point 1. As a result, the wall thickness of the tube at Point 1 formed under the current intensity of 1400 A is greater than that under 1200 A. The thickness of the thickening zone was the largest under the current intensity of 1400 A, with an average wall thickness of 5.602 mm and a thickening rate of 280%, and the smallest under 800 A, with an average wall thickness of 2.98 mm and a thickening rate of 149%.

**Figure 8:**
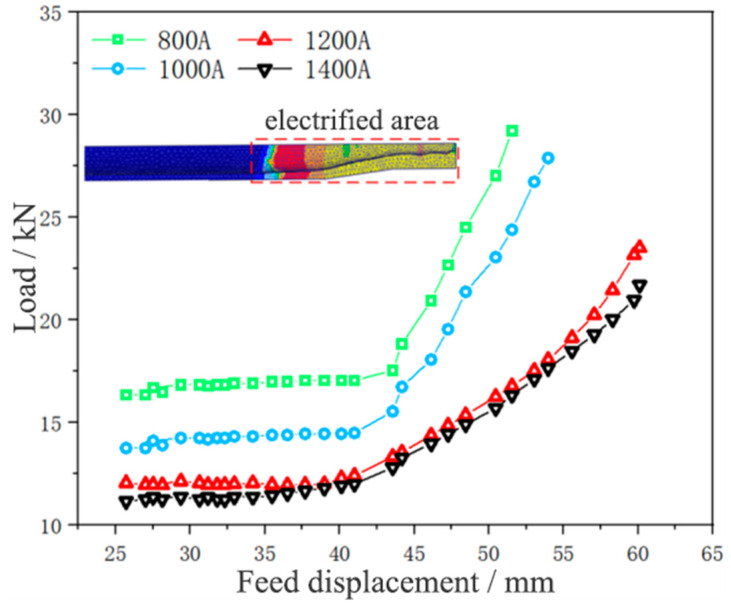
Displacement-load curves of tube thickening under different current intensities.

**Figure 9:**
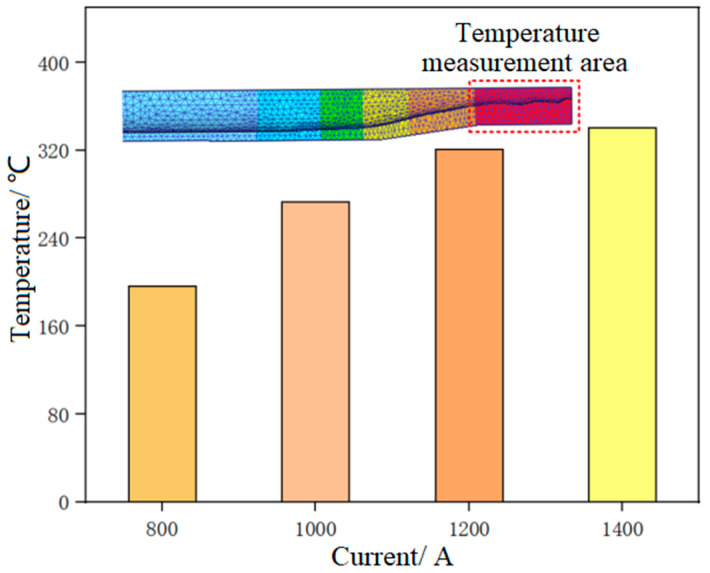
The thickening temperature of the tube under different current intensities.

Figure 8 and Figure 9 show the thickening displacement-load curve and temperature distribution of the 5A02 aluminum alloy tube under different current intensities, respectively.

After completing the necking, a straight section began to form at the front end of the necking tube. As the tube diameter and neck deformation length remained basically unchanged, it could be observed that the displacement-load curve of the tube was kept in a horizontal state during straight section forming, and the forming load of the tube was basically unchanged at this time. When the feed displacement approached 45 mm, the formation of the straight section at the front end of the necking was completed. The end face of the straight section tube contacted the thickened surface of the concave die, and the tube began to thicken. The material slowly accumulated and thickened, causing the load curve to rise sharply. As the thickening progressed, the load continued to increase. Generally speaking, the displacement-load curve showed a parallel upward trend with the decrease of current intensity, and the temperature showed an upward trend with the increase of current intensity. The current intensity of 800 A led to the largest forming load, the lowest temperature, the greatest difficulty in forming, and the worst effect. The current intensity of 1400 A led to the smallest forming load, the highest temperature, the easiest forming, and the best effect. When the current intensity grew from 800 A to 1200 A, the load decreased obviously. When the current intensity rose from 1200 A to 1400 A, the load decreased weakly. This is because when the current intensity rises from 800 A to 1200 A, the temperature gradient is larger, and the plasticity of the tube blank is obviously improved. When the current intensity rises from 1200 A to 1400 A, the temperature increase is smaller, and the plasticity improvement of the tube blank is weaker. It can be seen that in the process of tube thickening forming, a larger current intensity can reduce the load and is more conducive to forming, so the current intensity for tube thickening forming was selected as 1400 A in this study.

### 3.2. Effect of Feed Speed

#### 3.2.1. Effect of Feed Speed on Necking Forming

Similarly, the effect of feed speed on the necking forming of the 5A02 aluminum alloy tube was analyzed using the control variable method. As shown in Table 1, necking tests were performed under a fixed current intensity of 300 A and feed speeds of 2, 5, 10, and 20 mm/min.

**Figure 10:**
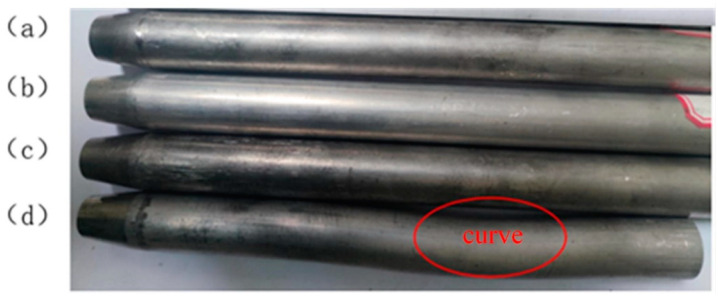
Necking parts of 5A02 aluminum alloy tube at different feed speeds: (**a**) v = 2 mm/min; (**b**) v = 5 mm/min; (**c**) v = 10 mm/min; (**d**) v = 20 mm/min.

Figure 10 shows the necking tube fittings formed at the feed speeds of 2, 5, 10, and 20 mm/min, respectively.

At a low feed speed (v ≤ 10 mm/min), the formed necking tube fittings showed good quality with no surface damage and no bending or instability in the supporting zone. When the feed speed was too high (v ≥ 20 mm/min), the quality of the formed necking tube was poor, and bending occurred in the supporting zone of the tube. This is because, with the increase in feed speed, the necking formation of the tube becomes faster. When the low-temperature material flows into the necking zone from the supporting zone, the temperature of the low-temperature material fails to rise high enough under the action of current within enough time, and the plasticity is insufficient, which leads to an increase in the forming load and difficulty, so bending occurs. Therefore, the suitable feed speed for tube necking deformation should not be greater than 10 mm/min.

**Figure 11:**
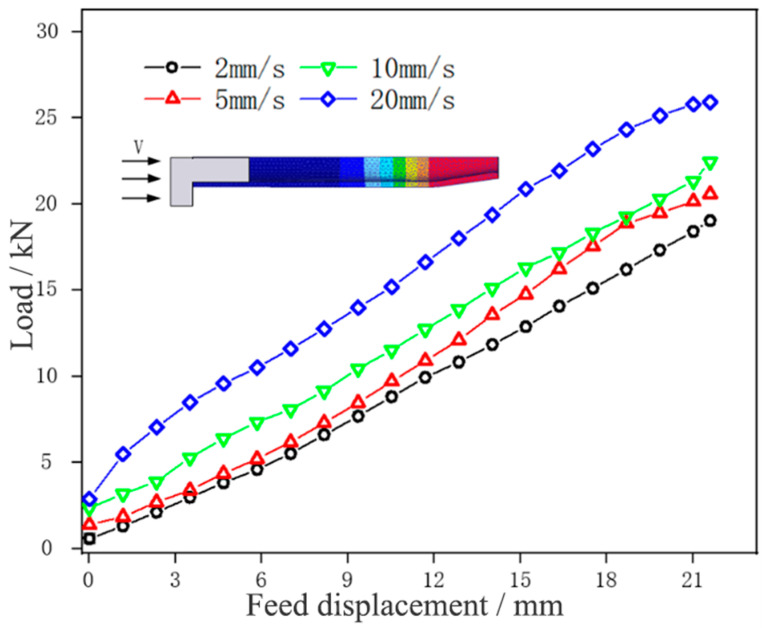
Displacement-load curves of tube necking under different feed speeds.

Figure 11 shows the load change of tube necking at different feed speeds.

It could be seen that the forming load curve of the tube blank at different feed speeds was distributed in an oblique straight line and parallel, and the load generally increased with the increase in feed speed. At a feed speed of 2 mm/min, the necking forming load of the tube blank was the lowest, at 14.28 kN. With the slow increase of feed speed (v ≤ 10 mm/min), the necking forming load of the tube blank grew slightly, and with the step increase of speed, the load increment also increased slowly, mainly because the accelerated feed speed aggravated the severity of tube deformation, accelerated the strain rate of the tube, and intensified the difficulty of tube deformation. When the feed speed was 20 mm/min, the necking forming load of the tube blank was the highest, being 20.72 kN, and the forming load curve rose greatly. This is because the tube in the necking zone lacks enough time to heat up due to the step increase in feed speed, the tube blank is not plastic enough, and thus a larger forming load is needed to complete necking forming. In the process of tube necking, therefore, the load can be reduced by choosing a smaller feed speed, which is more conducive to forming. At the same time, the feed speed in the necking stage was selected at 10 mm/min, aiming to reduce the time cost.

#### 3.2.2. Effect of Feed Speed on Thickening Forming

The experimental scheme adopted is shown in Table 1. The effect of different feed speeds (0.2, 1, 2, and 5 mm/min) on the thickening forming of the 5A02 aluminum alloy tube fitting under a fixed current intensity of 1400 A was explored.

**Figure 12:**
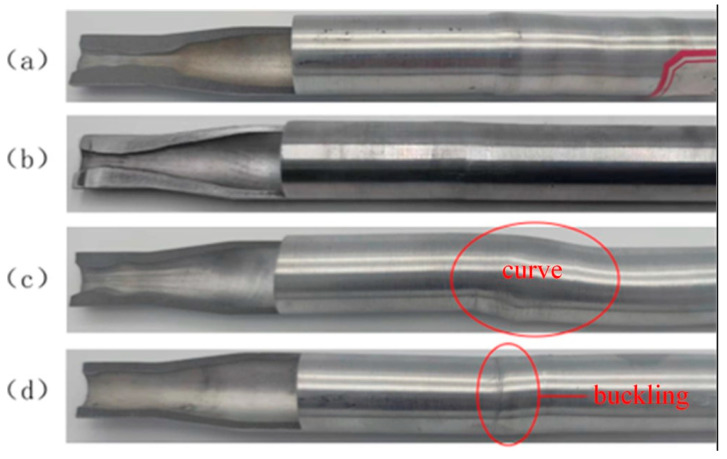
Thickening parts of 5A02 aluminum alloy tube at different feed speeds: (**a**) v = 0.2 mm/min; (**b**) v = 1 mm/min; (**c**) v = 2 mm/min; (**d**) v = 5 mm/min.

Figure 12 shows a thickened tube formed at different feed speeds.

Figure 12a displays the thickened tube fitting formed at the feed speed of 0.2 mm/min. The thickening forming was the most obvious after the straight section forming was completed. The wall thickness of the thickening zone met the target requirements, and the tube fitting was not subjected to buckling, bending, or other instability phenomena. Figure 12b shows the thickened tube fitting formed at the feed speed of 1 mm/min. After the straight section forming was completed, the thickening forming effect was slightly weaker than that in Figure 12a. The wall thickness of the thickening zone met the target requirements, and the tube fitting experienced no buckling, bending, or other instability phenomena. Figure 12c exhibits the thickened tube fitting formed at the feed speed of 2 mm/min. After the straight section forming was completed, the thickening forming effect was poor, the wall thickness of the thickening zone failed to meet the target requirements, and bending took place in the supporting zone of the tube fitting. Figure 12d shows the thickened tube fitting formed at the feed speed of 5 mm/min. After the straight section forming was completed, the thickening forming effect was the worst; the wall of the thickening zone was almost not thickened, and bending occurred in the supporting zone of the tube fitting. This is because when the feed speed of the tube exceeds 2 mm/min, the thickening forming speed of the tube is relatively too fast, and the material flows into the thickening zone from the low-temperature necking zone, so the temperature of the low-temperature material fails to rise high enough under the action of current within enough time, resulting in insufficient plasticity and leading to an increase in the forming load, which then exceeds the limit that the tube in the supporting zone can bear, thus resulting in bending.

**Figure 13:**
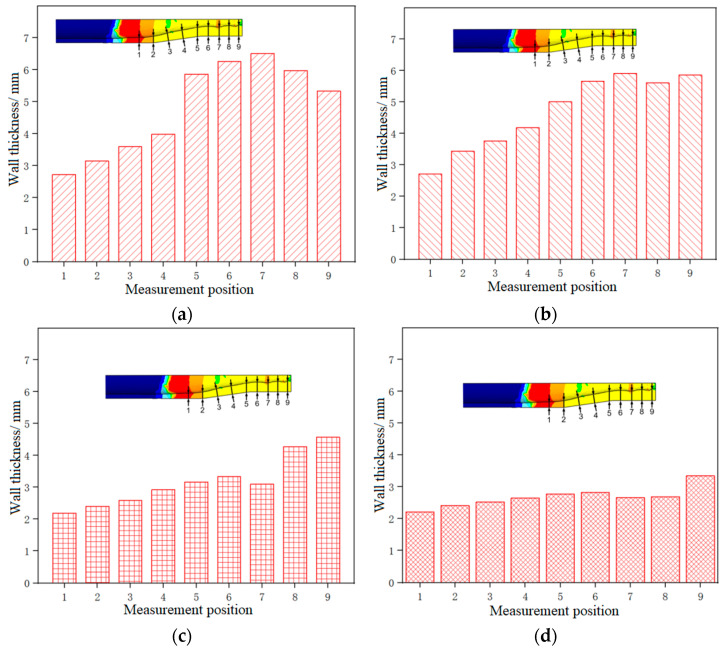
Wall thickness distribution of 5A02 aluminum alloy tube thickening parts at different feed speeds: (**a**) v = 0.2 mm/min; (**b**) v = 1 mm/min; (**c**) v = 5 mm/min; (**d**) v = 10 mm/min.

Figure 13 shows the wall thickness distribution of the formed thickened tube under different current intensities. It could be seen that the wall thickness of the tube thickening zone decreased with the increase of the feed speed, and when the feed speed exceeded 5 mm/min, the tube blank hardly thickened.

This is because under the same current intensity, the slower the feeding speed of the tube blank, the longer the heating time of the material, the higher the temperature, the better the plasticity, the better the fluidity of the material, the easier the thickening forming, and the more the materials accumulated in the thickening zone, and the thicker the wall thickness of the thickening zone. On the contrary, the faster the feeding speed of the tube blank, the shorter the heating time of the material, the lower the temperature, the worse the plasticity, the more intense the deformation, the more difficult the forming, and the less the materials accumulated in the thickening zone. However, the thickness of the necking zone and the end of the supporting zone of the tube blank first increased and then decreased with the decrease in feed speed. This is because as thickening forming proceeds, some materials do not flow to the thickening zone but accumulate in the necking zone and the supporting zone and this part of materials increases with the increase of the feed speed. At the feed speed of 1 mm/min, the tube with the feed speed of 1 mm/min achieved the best thickening effect, followed by the tube with the feed speed of 2 mm/min and the tube with the feed speed of 5 mm/min successively, and the amount of feed was ranked in the same order. When the feed speed was 1 mm, the lower the feed speed, the better the fluidity of the material. Under the same feed speed, more materials flew to the thickening zone of the tube blank, and less material accumulated in other zones. The wall thickness of the thickening zone of the tube was the largest at the feed speed of 0.2 mm/min, with an average wall thickness of 5.984 mm and a thickening rate of 303.2%. When the feed speed was 5 mm/min, the wall thickness was the smallest, with an average wall thickness of 2.856 mm and a thickening rate of 142.8%.

**Figure 14:**
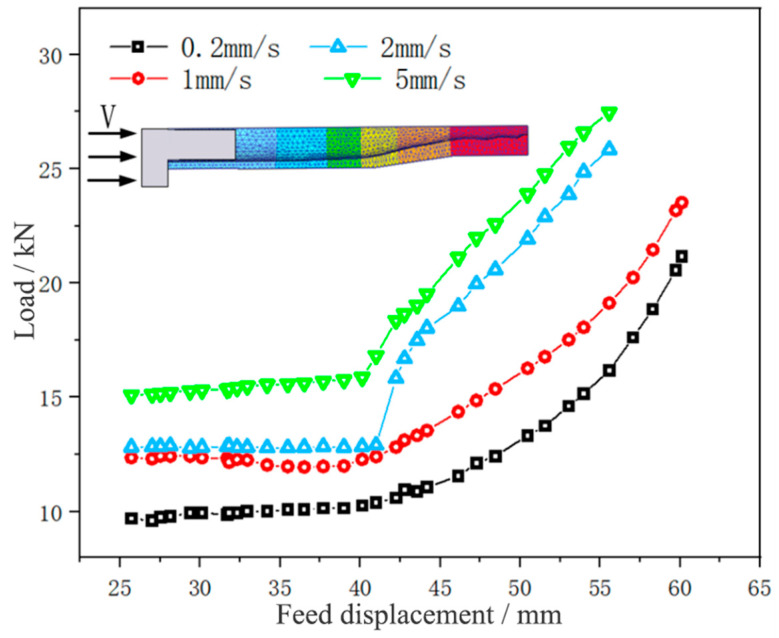
Displacement-load curves of 5A02 aluminum alloy tube thickening at different feed speeds.

Figure 14 shows the thickening displacement-load curve of the 5A02 aluminum alloy tube at different feed speeds.

It could be observed that the forming load of the tube blank grew with the increase of the feed speed, and the thickening forming load curve was convex at the feed speed of 2 and 5 mm/min and concave at the feed speed of 0.2 and 1 mm/min. This is because the thickening forming load curve at the feed speed of 2 and 5 mm/min does not completely describe the thickening forming load of the tube, and there are also loads such as buckling and bending of the tube blank. Thus, it can be seen that in the process of tube thickening forming, a smaller feed speed can reduce the load and is more conducive to forming. In this study, the feed speed for thickening forming was chosen as 1 mm/min to reduce the time cost while meeting the experimental conditions.

## 4. Conclusions

1. During the necking forming stage, as the current intensity increases, the temperature of the tube blank in the necking area increases and the forming load decreases; The forming load generally increases with the increase of feed speed.
2. In the thickening forming stage, the displacement-load curve shows a parallel upward trend with the decrease of current intensity, while the temperature shows an upward trend with the increase of current intensity; The smaller feed speed can reduce the load and be more conducive to forming.
3. Increasing the temperature gradient difference between the thickening zone, necking zone, and support zone of the tube ensures that the initial forming load of the thickening forming is much lower than the bearing limit of the tube support zone, which is conducive to the thickening forming of the tube and thereby improves the thickening rate.
4. The process window for necking and thickening of the tube is obtained to manufacture good parts, that is, during the necking stage, the current intensity should not be less than 300 A and the feed speed should not exceed 10 mm/min; During the thickening stage, the current intensity should not be less than 1400 A and the feed speed should not exceed 1 mm/min. The establishment of a phased forming window has optimized the necking and thickening forming process, achieving precise control in stages, reducing energy loss, and improving efficiency.

## Figures and Tables

**Table 1:** Experimental scheme for electrically assisted necking and thickening.

Forming Stage	Experimental Parameters	Current Intensity	Feed Speed
Necking stage	Current intensity	100 A	10 mm/min
		200 A	
		300 A	
		400 A	
	Feed speed	300 A	5 mm/min
			10 mm/min
			20 mm/min
			50 mm/min
Thickening stage	Current intensity	800 A	1 mm/min
		1000 A	
		1200 A	
		1400 A	
	Feed speed	1400 A	0.2 mm/min
			1 mm/min
			2 mm/min
			5 mm/min

## Data Availability

Data are contained within the article.
